# Micro-simulation insights into the functional and mechanistic understanding of glycyrrhizin against asthma

**DOI:** 10.3389/fphar.2023.1220368

**Published:** 2023-08-28

**Authors:** Jian-Hong Qi, Dong-Chuan Xu, Xiao-Long Wang, Ding-Yuan Cai, Yi Wang, Wei Zhou

**Affiliations:** ^1^ Department of Pharmaceutics, China Pharmaceutical University, Nanjing, China; ^2^ Shandong Academy of Traditional Chinese Medicine, Jinan, China; ^3^ Shandong University of Traditional Chinese Medicine, Jinan, China; ^4^ Key Laboratory of Traditional Chinese Medicine Classical Theory, Ministry of Education, Shandong University of Traditional Chinese Medicine, Jinan, China; ^5^ Shandong Provincial Key Laboratory of Traditional Chinese Medicine for Basic Research, Shandong University of Traditional Chinese Medicine, Jinan, China

**Keywords:** glycyrrhizin, asthma, transgelin-2, mechanism, simulation

## Abstract

Asthma is a common chronic respiratory disease, which causes inflammation and airway stenosis, leading to dyspnea, wheezing and chest tightness. Using transgelin-2 as a target, we virtually screened the lead compound glycyrrhizin from the self-built database of anti-asthma compounds by molecular docking technology, and found that it had anti-inflammatory, anti-oxidative and anti-asthma pharmacological effects. Then, molecular dynamics simulations were used to confirm the stability of the glycyrrhizin-transgelin-2 complex from a dynamic perspective, and the hydrophilic domains of glycyrrhizin was found to have the effect of targeting transgelin-2. Due to the self-assembly properties of glycyrrhizin, we explored the formation process and mechanism of the self-assembly system using self-assembly simulations, and found that hydrogen bonding and hydrophobic interactions were the main driving forces. Because of the synergistic effect of glycyrrhizin and salbutamol in improving asthma, we revealed the mechanism through simulation, and believed that salbutamol adhered to the surface of the glycyrrhizin nano-drug delivery system through hydrogen bonding and hydrophobic interactions, using the targeting effect of the hydrophilic domains of glycyrrhizin to reach the pathological parts and play a synergistic anti-asthmatic role. Finally, we used network pharmacology to predict the molecular mechanisms of glycyrrhizin against asthma, which indicated the direction for its clinical transformation.

## 1 Introduction

Asthma is a common inflammatory disease of the airways that involves multiple types of cells and cellular components (Papi et al., 2018). In China, the prevalence of asthma was about 4.2% (Brusselle and Ko, 2019), with a higher incidence in children (Chang, 2012). It was characterized by intermittent breathing difficulties, wheezing, and coughing, which severely impacted patients’ quality of life (To et al., 2012). If left untreated, acute asthma attacks can result in death due to respiratory distress (Fergeson et al., 2017). At present, corticosteroids and beta-2 agonists were commonly used to treat asthma, but their efficacy varied significantly among individuals and long-term use can lead to side effects, which limited their use (Kersten et al., 2017; Heffler et al., 2018; Miller et al., 2021). Targeted therapy was a treatment that targeted the identified pathogenic parts at the molecular level. With the development of molecular biotechnology, targeted drugs for asthma have been developed in recent years (Papi et al., 2018).

Transgelin-2 was widely distributed in the human body, and had a variety of biological functions, including muscle contraction, cell migration, cell proliferation and apoptosis (Yin et al., 2019). It mainly existed in smooth muscle cells, such as vascular smooth muscle cells, bronchial smooth muscle cells and gastrointestinal smooth muscle cells, and other cell types, such as neurons, cardiomyocytes and renal tubular cells. It can regulate the contraction and relaxation of smooth muscle cells by binding to actin. In addition, the expression of transgelin-2 can also be detected in tumor cells. Studies have shown that it can promote the proliferation and invasion of tumor cells and is related to the prognosis of tumors (Kim H. R. et al., 2021).

Researchers demonstrated the critical role of metallothionein-2 (MT-2) in the pathogenesis of asthma using a gene-knockout mouse model. Then, they identified transgelin-2 as the receptor for MT-2 on the bronchial smooth muscle. Furthermore, they confirmed transgelin-2 as a new target for anti-asthmatic therapy, using some techniques such as laser confocal microscopy, RNA interference, surface plasmon resonance, and gene-knockout animal models (Yin et al., 2018). Additionally, the lead compounds specifically binding to transgelin-2 were screened from 6,000 compounds using molecular docking techniques. Through cellular biology and a clinically relevant model of asthma, they validated that the lead compound TSG12, which relaxed the bronchial smooth muscle, had promising clinical applications as a potential anti-asthmatic drug, and they demonstrated that activation of the transgelin-2 led to bronchial smooth muscle relaxation via the “calcium sensitization” pathway (Yuan et al., 2022).

Currently, there were few active targets for asthma treatment, and the discovery of new targets and the development of small molecule drugs were particularly important. Based on this situation, this study efficiently screened potential active compounds targeting transgelin-2 and predicted the biological functions and signaling pathways for anti-asthma effects. At the same time, in-depth structural biology analysis and pharmaceutical studies were conducted on the lead compound to provide important references for the clinical translation of anti-asthma small molecule drugs.

## 2 Materials and methods

### 2.1 Establishment of anti-asthmatic compound database

We retrieved 1,504 unique compounds using “Asthma” as a keyword in TCMSP (https://old.tcmsp-e.com/) database (Ru et al., 2014), and downloaded their MOL2 structure files. Then, we prepared the compounds using the Raccoon script and converted the MOL2 to PDBQT format for high-throughput molecular docking.

### 2.2 High-throughput screening of transgelin-2 agonist

It was known that transgelin-2 was an important target for improving bronchial smooth muscle and treating asthma, and TSG12 has been identified as its positive ligand. Based on this, we downloaded the three-dimensional crystal structure of transgelin-2 (PDB ID: 1WYM) from RCSB PDB (https://www.rcsb.org/) database (Berman et al., 2000; Yin et al., 2018). We used AutoDockTools and PyMol software to view the protein structure and found that the CH domain of transgelin-2 contains 155 amino acids and 20 conformations, of which Model 5 was considered to be the active conformation (Yin et al., 2018). Therefore, we used this conformation for subsequent experiments. Firstly, we prepared the protein based on its structural characteristics: adding hydrogen, computing Gasteiger charges, assigning AutoDock 4 (AD4) atomic types, and merging 902 non-polar hydrogen atoms. Then, the active pocket was selected with TSG12 as the center, and the center coordinates (−0.926, 13.178, −2.446) were determined. We set the exhaustiveness of the pocket to 8, the energy range between molecules to 3, and the number of conformations to 9. Finally, AutoDock Vina and python scripts were used for high-throughput screening of lead compounds in the anti-asthmatic compound database (Trott and Olson, 2010).

### 2.3 Computational modeling

GROMACS (Groningen Machine for Chemical Simulations) is a software package used for molecular dynamics simulations, known for its high computational speed and extensive features (Van et al., 2005). In this section, we explored the stability of the complex formed by transgelin-2 and the lead compound, using TSG12 as the positive group. The topology and structure files of the lead compound was prepared by PRODRG (http://davapc1.bioch.dundee.ac.uk/cgi-bin/prodrg) website (Gopal et al., 2015). GROMOS96 43a1 force field was used based on the system characteristics, and a dodecahedron box was constructed (Qi et al., 2022a). The simple point charge (SPC) water model was defined and ions were added to ensure the system’s electrical neutrality (Gopal et al., 2015). The steepest descent minimization method was used to achieve energy minimization and ensure the system convergence. The system conditions were set: constant temperature (298 K) and pressure (1 bar) (Gauden et al., 2014). Particle mesh Ewald (PME) algorithm was used to calculate long-range electrostatic interactions, while the cut-off algorithm was used for short-range interactions (Rout and Mahapatra, 2016). Finally, the mdrun command was used to perform a 100 ns simulation.

To further develop the application of lead compounds in the field of Pharmaceutics, we conducted self-assembly simulations of lead compounds in an aqueous environment. Firstly, topology, structure, and force field files were downloaded from the ATB (http://atb.uq.edu.au/) website (Malde et al., 2011). Using GROMACS, a cubic box with a side length of 6 nm was constructed, 10 lead compounds were randomly inserted into the box, and the SPC water model was built. The remaining processes were the same as described above. Finally, the mdrun command was used for a 50 ns simulation. In addition, salbutamol is a representative drug for treating asthma (Marques and Vale, 2022). Evidence of the synergy between the lead compound and salbutamol in treating asthma was obtained through literature research and molecular dynamics simulation was used to get the functional and mechanistic understanding involved.

### 2.4 Exploration of molecular mechanisms

Network pharmacology refers to the interdisciplinary application of systems biology, network science, pharmacology, and other fields to study the molecular mechanisms of drug and guide the design and development of new drugs (Zhang et al., 2019). Firstly, the lead compound was imported into the TCMSP database to obtain the MOL2 format files, which were then imported into the PharmMapper (http://lilab-ecust.cn/pharmmapper/) online database for target prediction (Wang et al., 2017). In addition, asthma-related targets were searched in the Drugbank (https://go.drugbank.com/), TTD (https://db.idrblab.net/ttd/), and Genecards (https://www.genecards.org/) databases (Rebhan et al., 1997; Wishart et al., 2018; Wang et al., 2020). The intersection of compound-targets and disease-targets was taken to identify the potential targets of the lead compound. The intersection targets were then imported into the STRING (https://cn.string-db.org/) database for protein-protein interaction (PPI) analysis (Szklarczyk et al., 2019), and the results were exported in TSV format and visualized using the Cytoscape 3.6.1 software (Shannon et al., 2003). Furthermore, the intersection targets were imported into the Metascape (https://metascape.org/) database for Gene Ontology (GO) and Kyoto Encyclopedia of Genes and Genomes (KEGG) analysis to enrich and annotate the biological information and signaling pathways related to the lead compound (Zhou et al., 2019).

## 3 Results and discussion

### 3.1 Discovery of glycyrrhizin as a transgelin-2 agonist

**FIGURE 1:**
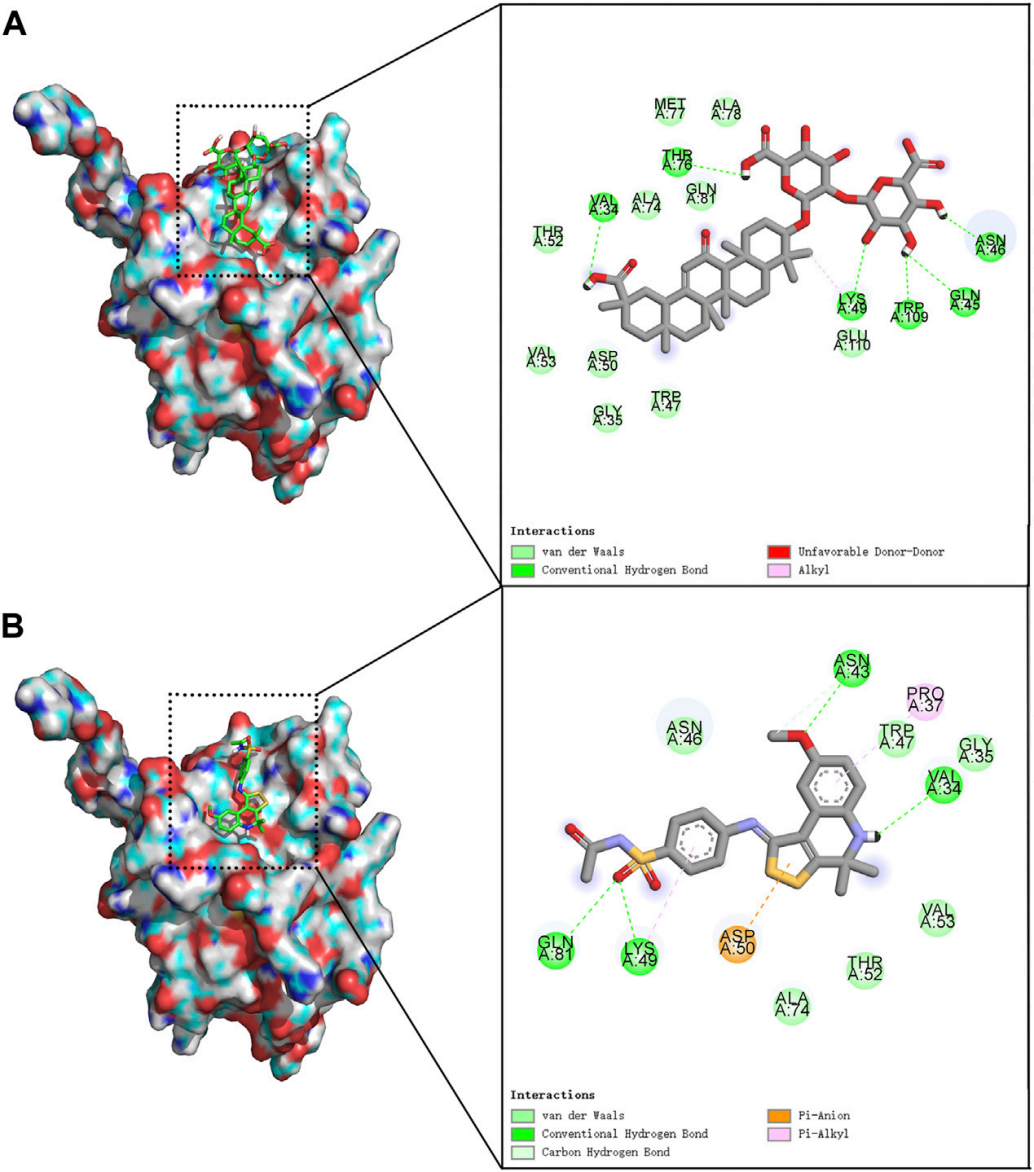
Molecular docking model. ***Notes*:** The left side was 3D models and the right side was 2D models. **(A)** glycyrrhizin-transgelin-2. **(B)** TSG12-transgelin-2.

We screened 17 compounds from the anti-asthmatic compound database that scored better than the original agonist TSG12 (Table 1). Among them, glycyrrhizin has been reported to have pharmacological effects such as anti-inflammatory, antioxidant, immune regulation, and anti-asthmatic (Yang et al., 2010; Richard, 2021). Docking studies revealed that glycyrrhizin and TSG12 occupied the same active pocket (Figure 1), but glycyrrhizin bound more strongly to the active site. Here, we found that there were three interactions between glycyrrhizin and TSG12: hydrogen bond, van der Waals interaction and hydrophobic interaction. Transgelin-2 residues Val^34^, Gln^45^, Asn^46^, Lys^49^, Thr^76^ and Trp^109^ could form hydrogen bonds with glycyrrhizin. There was van der Waals interaction between glycyrrhizin and residues Gly^35^, Trp^47^, Asp^50^, Thr^52^, Val^53^, Ala^74^, Met^77^, Ala^78^, Gln^81^ and Glu^110^. There was also hydrophobic interaction between a methyl group on the quintuple ring of glycyrrhizin and residue Lys^49^. In addition, the hydrophilic domains played a major role in the binding of glycyrrhizin to transgelin-2, which may be the key functional groups targeting transgelin-2. (Figure 1A). Compared with TSG12 (Figure 1B), van der Waals interaction contributed more to the binding energy between glycyrrhizin and transgelin-2, which was also determined by the structural characteristics. Importantly, residues Val^34^, Gly^35^, Asn^46^, Trp^47^, Lys^49^, Asp^50^, Thr^52^, Val^53^, Ala^74^ and Gln^81^ were important for the stability of glycyrrhizin-transgelin-2 and TSG12-transgelin-2 complexes, but the interactions involved were slightly different. Therefore, glycyrrhizin could improve asthma by targeting transgelin-2.

**TABLE 1:** The information of 17 compounds scored better than TSG12.

MOL ID	Name	CAS	AlogP	Scores (kcal/mol)
MOL004421	hexandraside D	♦ 137218-02-1	−0.09	−7.7
MOL004230	stylopine	♦ 4312-32-7	3.2	−7.6
MOL004231	tetrahydrocorysamine	♦ 32043-26-8	3.52	−7.6
MOL004932	glycyrrhizin	♦ 103000-77-7	2.42	−7.6
MOL009074	chitranone	♦ 58274-95-6	3.52	−7.5
MOL002662	rutaecarpine	♦ 84-26-4	3.36	−7.4
MOL003956	dihydrorutaecarpine	♦ 59863-00-2	3.31	−7.3
MOL004422	hexandraside E	*N/A*	−0.37	−7.3
MOL004820	kanzonols W	*N/A*	3.63	−7.3
MOL000781	PDSP1_000624	♦ 298-45-3	3.08	−7.2
MOL001423	isorhamnetin-3-o-beta-d-rutinoside	*N/A*	−1.19	−7.2
MOL005079	corilagin	♦ 23094-69-1	0.9	−7.2
MOL004401	bilobetin	♦ 521-32-4	4.61	−7.1
MOL005852	1,2,6-tri-O-galloyl-β-D-glucopyranoside	*N/A*	1.04	−7.1
MOL004383	yinyanghuo B	*N/A*	5	−7
MOL008644	tetrahydrocorysamine	♦ 32043-26-8	3.52	−7
MOL008646	6-acetonyldihydrosanguinarine	♦ 37687-34-6	3.43	−7

### 3.2 Complex simulation of glycyrrhizin and transgelin-2

**FIGURE 2:**
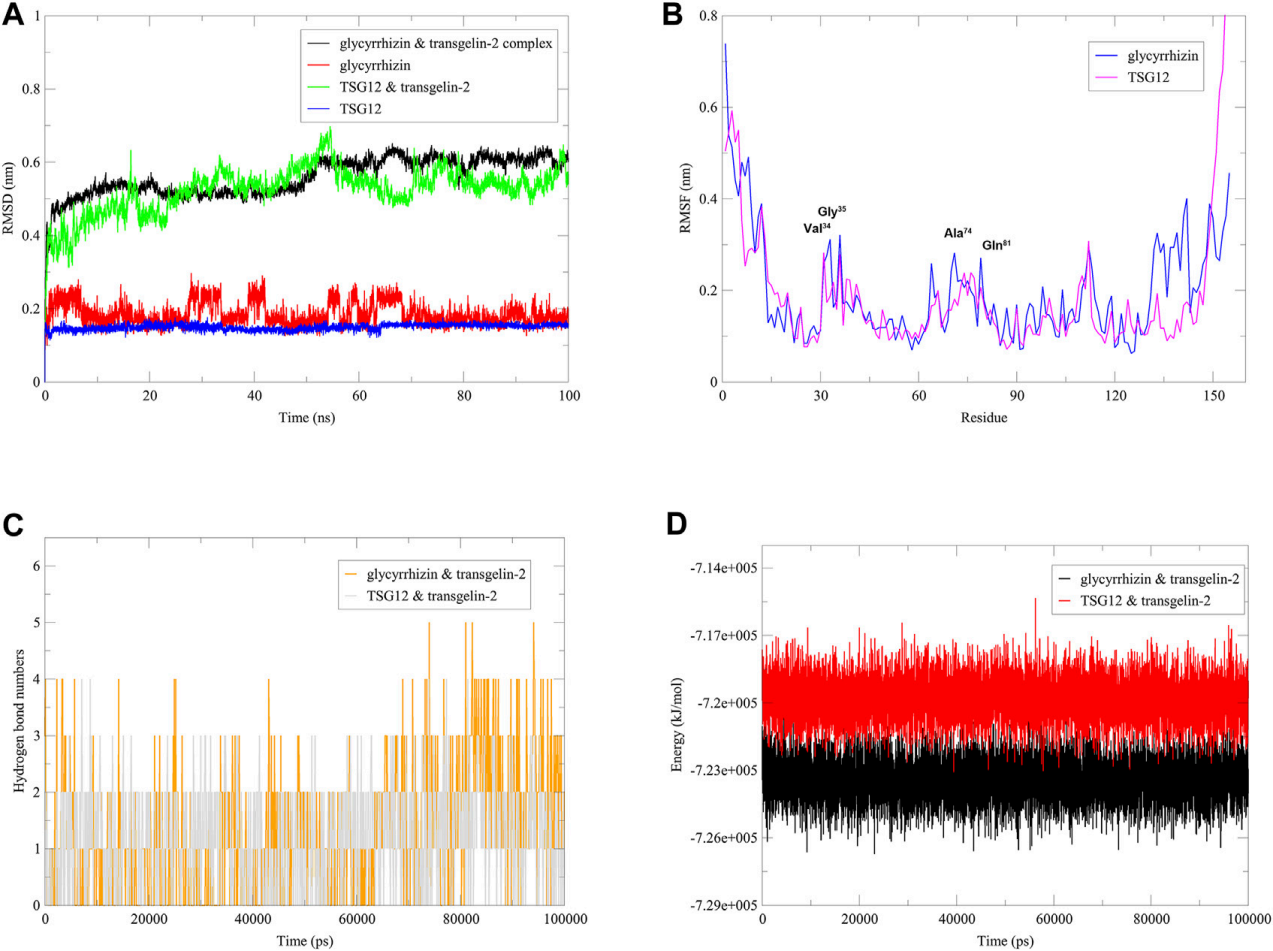
Dynamics simulation of complexes. ***Notes*: (A)** RMSD. **(B)** RMSF. **(C)** Hydrogen bond numbers. **(D)** Energy.

In order to further investigate the stability of the complex formed by glycyrrhizin and transgelin-2, and to provide strong evidence for glycyrrhizin as an agonist of transgelin-2 from a structural biology perspective, we conducted molecular dynamics simulations using the GROMACS software (Supplementary Figure S1.S2). Root Mean Square Deviation (RMSD) is a metric used to compare the similarity between two structures, which quantifies the differences in distances between corresponding atoms in the two structures. In the study of protein-ligand interactions, RMSD is often used to assess the matching degree and stability of the ligand-protein structure (Pitera, 2014; Qi et al., 2022b). As shown in Figure 2A, it can be observed that during the 100 ns simulation time, glycyrrhizin and TSG12 exhibited similar RMSD values and comparable fluctuation amplitudes, and they reached a stable state after 70 ns. Root Mean Square Fluctuation (RMSF) is used to describe the variation of each amino acid residue’s atoms relative to the average position. It can be used to assess the dynamic changes of each amino acid residue in the protein and analyze the flexibility of the protein (Su et al., 2022). TSG12 and glycyrrhizin exhibited differences in the perturbation of transgelin-2 structure, particularly in the residues 130–150. However, they both had similar and significant effects on residues Val^34^, Gly^35^, Ala^74^, and Gln^81^, which were consistent with the docking results, indicating that these sites played important roles in the activation of transgelin-2 (Figure 2B). Hydrogen bonds play a crucial role in the binding of proteins and compounds. In proteins, hydrogen bonds are one of the key forces that contribute to the tertiary structures (Bowie, 2011). Amino acid residues in proteins interact with each other or other molecules through hydrogen bonds during the folding process. The formation and breaking of hydrogen bonds regulate the structures of proteins, which determines their functions (Bolen and Rose, 2008). In compounds, hydrogen bonds can interact with amino acid residues in proteins, facilitating the binding of compounds to proteins. This interaction can affect the structure, stability, and activity of compounds, thereby influencing their biological effects (Liu et al., 2022). Thus, hydrogen bonds play a vital role in the binding of proteins and compounds, and are of great significance in understanding the structure and function of proteins and compounds (Chen et al., 2016). Here, we used geometric criteria to determine the hydrogen bond. When the hydrogen bond donor-acceptor distance was less than 3.5 Å and the angle between the two was less than 30°, it was considered to be a hydrogen bond (Qi et al., 2022b). As shown in Figure 2C, TSG12 and glycyrrhizin can bind to the receptor during the 100 ns simulation, with a similar number of hydrogen bonds. Additionally, their energies were very close (Figure 2D). Therefore, it was very likely that glycyrrhizin could activate the function of transgelin-2 by altering the structure, which was similar to TSG12.

### 3.3 Self-assembly simulation of glycyrrhizin

**FIGURE 3:**
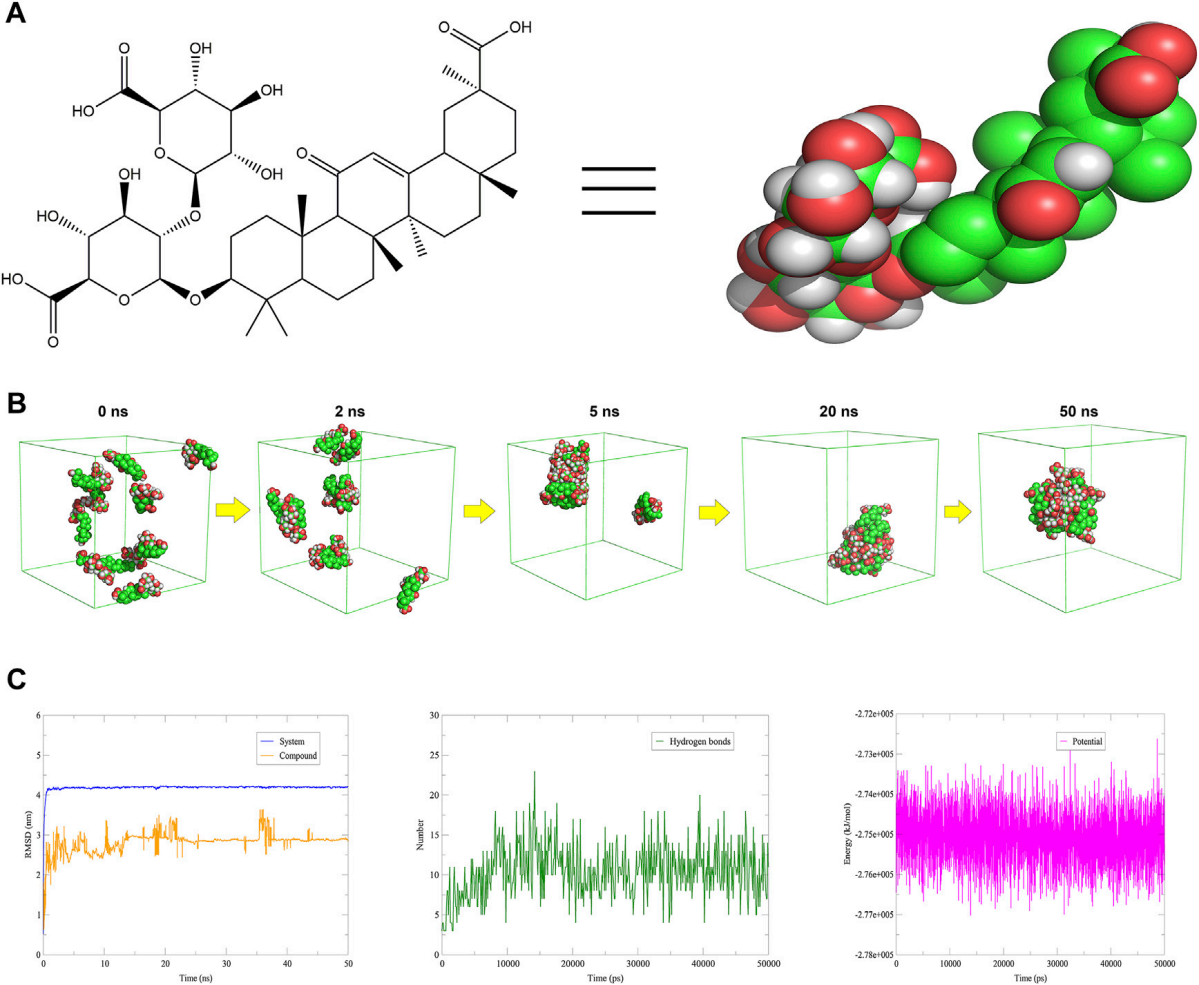
Self-assembly simulation of glycyrrhizin. ***Notes*: (A)** The structure of glycyrrhizin: the green sphere is C, the red sphere is O, and the white sphere is H. **(B)** Self-assembly simulation of 10 glycyrrhizin in aqueous solution. **(C)** Data analysis after simulation: RMSD, hydrogen bonds and energy.

Glycyrrhizin is a compound with amphiphilic properties (Figure 3A), which has been reported to self-assemble into nanoparticles under certain conditions (Kim A. V. et al., 2021). It was found that the critical aggregation concentration (CAC) value of glycyrrhizin was 2.38 mmol/L, indicating that it can self-assemble into nanoparticles at concentrations higher than CAC (Han et al., 2022). It has also been reported that dimeric complexes can be observed in solution at concentrations of 0.01–1 mmol/L, while large aggregates can be formed at concentrations greater than 1 mmol/L (Polyakov, 2011). The dimer of glycyrrhizin generated under low concentration conditions can bind hydrophobic molecules with its ring structure to form “host-guest complexes” and increase the solubility of the latter. Glycyrrhizin nanoparticles were generally formed under high concentration conditions and had high stability under medium or low pH conditions (Polyakov, 2011). Many studies have used the self-assembly behavior of glycyrrhizin to develop new drug delivery systems, which not only improved the solubility of hydrophobic drugs but also utilized the pharmacological properties of glycyrrhizin. After loading curcumin with glycyrrhizin nanoparticles, membrane permeability was improved, and the oral bioavailability of the drug was increased by 19 times (Zhang et al., 2018). After loading paclitaxel with glycyrrhizin nanoparticles, the absorption of the drug in the intestine was enhanced, and the oral bioavailability of the drug was increased by 6 times (Yang et al., 2015). After loading lutein and zeaxanthin with glycyrrhizin nanoparticles, the solubility of the drug *in vitro* was increased by 1,000 times, and the oxidative stability was enhanced (Apanasenko et al., 2015). These findings suggested that glycyrrhizin readily self-assembled into nanoparticles.

In this section, we constructed a 6 × 6 × 6 nm^3^ SPC water model box and added 10 glycyrrhizin molecules to investigate their self-assembly ability under certain conditions (Feng et al., 2018). Through self-assembly simulation experiments (Supplementary Figure S3), we obtained the mechanistic understanding of the nanoparticle formation: 10 glycyrrhizin molecules gradually aggregated within 50 ns and exhibited a pattern where hydrophilic domains were on the outside and most hydrophobic domains were on the inside (Figure 3B). In addition, the RMSD and energy of glycyrrhizin and its system tended to stabilize during nanoparticle formation, and hydrogen bonds were also one of the main driving forces besides hydrophobic interactions (Figure 3C). Therefore, we can utilize the amphiphilic and self-assembly properties of glycyrrhizin to design a low-toxic carrier for hydrophobic drugs that enhanced their solubility.

### 3.4 The functional and mechanistic understanding underlying the synergistic effects of glycyrrhizin and salbutamol against asthma

It was reported that glycyrrhizin had a synergistic effect on the anti-asthmatic properties of salbutamol (Yang et al., 2010). In addition, the combination of the two drugs has shown pharmacological effects of anti-inflammatory, immune regulation and inhibition of apoptosis induced by salbutamol. However, the functional and mechanistic understanding were unclear. We conducted computer simulation experiments (Supplementary Figure S4) based on the clinical dosages and compound ratios (Yang et al., 2010): 8.31 mmol/L salbutamol and 24.93 mmol/L glycyrrhizin. We constructed a cubic box with a side length of 13 nm and formed a SPC water model system, and then added 11 salbutamol and 33 glycyrrhizin molecules, respectively. The number of compounds was calculated according to the following equation:

$$N=C\times V\times N_{A}$$ (1)

where N is the amount of substance in moles, C is the concentration of the substance in solution, V is the volume of the solution, $N_{A}$ is Avogadro constant.

**FIGURE 4:**
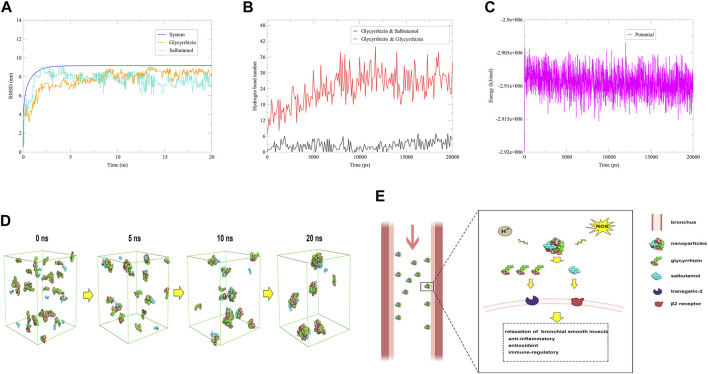
Mechanistic simulation underlying the synergistic effects of glycyrrhizin and salbutamol against asthma. ***Notes*: (A)** RMSD. **(B)** Hydrogen bond numbers. **(C)** Energy. **(D)** Self-assembly simulation of 33 glycyrrhizin and 11 salbutamol in aqueous solution. **(E)** Possible mechanism underlying the synergistic effects of glycyrrhizin and salbutamol against asthma.

The RMSD of the system, glycyrrhizin and salbutamol reached a plateau after about 2.5 ns (Figure 4A). The amount of hydrogen bonds between glycyrrhizin and glycyrrhizin reached a stable state after 7.5 ns, which may be related to the participation of salbutamol (Figure 4B). In addition, the average energy of the system was −2.91 × 10^−6^ kJ/mol, which was significantly lower than that of the glycyrrhizin self-assembly system (Figure 4C). This indicated that the salbutamol could accelerate the formation of nanoparticles and improve the stability of the system. As shown in Figure 4D, the majority of salbutamol adhered to the surface of glycyrrhizin nanoparticles through hydrogen bonding and hydrophobic interactions, forming a new nano-drug delivery system (NDDS). Because the concentration of salbutamol was significantly lower than that of glycyrrhizin, most of the hydrophilic regions of glycyrrhizin were still exposed to water. As mentioned above, these hydrophilic regions were key functional groups targeting transgelin-2 (Figure 1A). Therefore, it was speculated that salbutamol may target transgelin-2 through the hydrophilic regions of glycyrrhizin, and this NDDS can deliver most of the salbutamol to the bronchial smooth muscle. As the bronchial smooth muscle of asthmatic patients was exposed to a microenvironment characterized by ROS and subacidity, both of which can disrupt non-covalent interactions between molecules (Ricciardolo et al., 2004; Michaeloudes et al., 2022), it may be more conducive to the dispersion of nanoparticles at this part and the release of drugs. Once the self-assembled nanoparticles arrived at this part, it released glycyrrhizin and salbutamol through the special microenvironment. Since glycyrrhizin had pharmacological activities such as anti-inflammatory, antioxidant, and anti-asthmatic effects, it can work synergistically with salbutamol to treat asthma (Yang et al., 2010; Richard, 2021). It was known that the NDDS of glycyrrhizin also had significant liver-targeting function (El-Marakby et al., 2017; Zhao et al., 2021), so inhalation administration was a promising way for the delivery system to exert its therapeutic effect (Figure 4E).

### 3.5 Molecular mechanisms of glycyrrhizin against asthma

**FIGURE 5:**
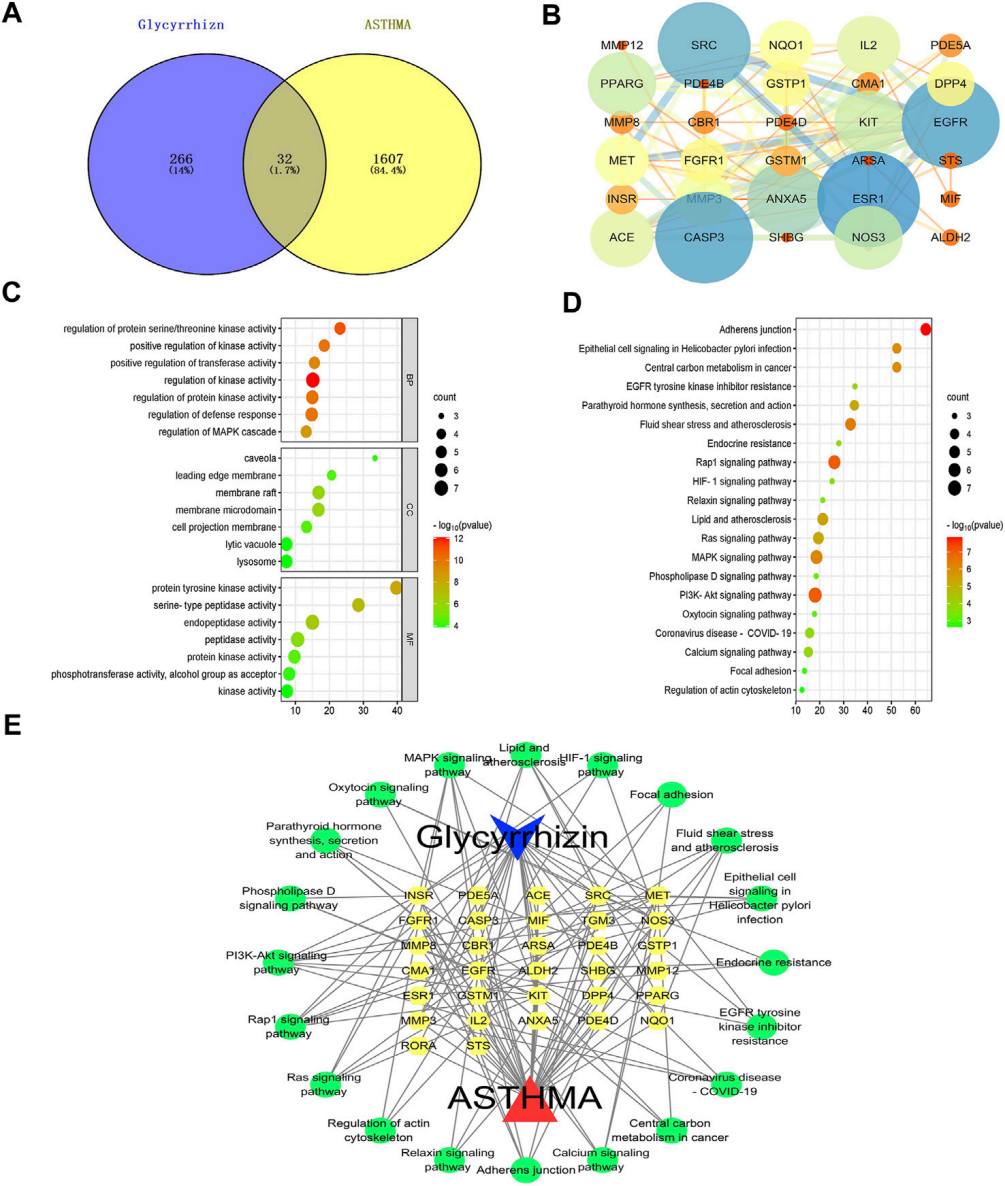
Molecular mechanisms of glycyrrhizin against asthma. ***Notes*: (A)** Venn diagram of the intersection target of glycyrrhizin and asthma. **(B)** PPI network: The larger the node, the darker the color, indicating that the node is more important; the thicker the edge, the darker the color, indicating that the edge is more important. **(C)** GO analysis: BP, CC and MF. **(D)** KEGG analysis. **(E)** Ingredient-target-signaling pathway network: the blue is ingredient, the yellow is the target, the green is the signaling pathway, and the red is disease.

To explore the molecular mechanisms underlying the anti-asthmatic effects of glycyrrhizin, we conducted network pharmacology analysis on public databases. Firstly, we collected 299 glycyrrhizin-related targets and 1,639 asthma-related targets, and mapped them to identify 33 overlapping targets (Figure 5A). By analyzing the protein-protein interaction relationship, we determined the importance of the targets in treating asthma. As shown in Figure 5B, the degree values of ESR1, EGFR, CASP3, and SRC were all greater than or equal to 15, highlighting their significance in the treatment of asthma with glycyrrhizin (Table 2). Asthma was characterized by pathological processes such as airway remodeling, inflammation, and excessive mucus secretion. Studies have reported an association between ESR1 mutant and asthma, particularly in female asthma patients, and suggested that it may worsen asthma progression by affecting airway remodeling (Dijkstra et al., 2006). Ovalbumin was a commonly used reagent to induce asthma in experiments, which can lead to abnormally high expression of CASP3 and increase inflammatory cells (Diao et al., 2016). A key factor in the pathological process of asthma is EGFR, which can disrupt the integrity of the claudin1-mediated epithelial barrier and exacerbate mucus secretion (Jia et al., 2021). In addition, SRC was closely related to the hypertrophy and proliferation of airway smooth muscle, possibly through the SRC/EGFR signaling pathway, which promoted the secretion of eosinophilic granulocytes (Krymskaya et al., 2005; Deng et al., 2022). These findings suggested that inhibiting SRC/EGFR may be a feasible strategy for treating asthma.

**TABLE 2:** The parameters of targets in PPI network (top 10).

Name	BetweennessCentrality	ClosenessCentrality	Degree
ESR1	0.34829523	0.69047619	17
EGFR	0.13774684	0.65909091	16
CASP3	0.09310523	0.64444444	16
SRC	0.18768393	0.64444444	15
ANXA5	0.03274103	0.59183673	12
PPARG	0.01877812	0.52727273	10
NOS3	0.02511596	0.55769231	10
KIT	0.02151361	0.55769231	10
ACE	0.03899303	0.53703704	9
IL2	0.02121648	0.50877193	9

Next, we performed biological information enrichment and annotation on these targets, ranking them in ascending order based on *p*-values. 417 biological processes (BP), 31 cellular components (CC), and 48 molecular functions (MF) were obtained by GO analysis. BP, such as regulation of kinase activity, regulation of protein kinase activity, regulation of defense response, regulation of protein serine/threonine kinase activity, positive regulation of kinase activity, positive regulation of transferase activity, and regulation of MAPK cascade. CC, such as membrane raft, membrane microdomain, cell projection membrane, leading edge membrane, caveola, lytic vacuole, and lysosome. MF, such as endopeptidase activity, peptidase activity, protein tyrosine kinase activity, protein kinase activity, phosphotransferase activity, alcohol group as acceptor, kinase activity, and serine-type peptidase activity (Figure 5C, Supplementary Table S1). 39 signaling pathways were obtained by KEGG analysis, such as PI3K-Akt signaling pathway, rap1 signaling pathway, MAPK signaling pathway, adherens junction, ras signaling pathway, fluid shear stress and atherosclerosis, lipid and atherosclerosis, epithelial cell signaling in *Helicobacter pylori* infection, central carbon metabolism in cancer, calcium signaling pathway, coronavirus disease-COVID-19, parathyroid hormone synthesis, secretion and action, EGFR tyrosine kinase inhibitor resistance, endocrine resistance, HIF-1 signaling pathway, relaxin signaling pathway, phospholipase D signaling pathway, oxytocin signaling pathway, focal adhesion, and regulation of actin cytoskeleton (Figure 5D, Supplementary Table S2). Additionally, we constructed a network of ingredient-target-signaling pathway to provide a comprehensive understanding of the molecular mechanisms of glycyrrhizin in treating asthma (Figure 5E, Supplementary Table S3).

## 4 Conclusion

In this study, we established an anti-asthmatic compound database, using TSG12 as the positive group, and identified 17 compounds with docking scores superior to TSG12. Among them, glycyrrhizin has been reported to have pharmacological effects such as anti-inflammatory, antioxidant, immune-regulatory, and anti-asthmatic. The molecular docking results revealed that the hydrophilic domain played a major role in the binding between glycyrrhizin and transgelin-2, which may be a crucial functional group for targeting transgelin-2. Additionally, residues Val^34^, Gly^35^, Asn^46^, Trp^47^, Lys^49^, Asp^50^, Thr^52^, Val^53^, Ala^74^ and Gln^81^ were found to be important for the stability of the glycyrrhizin-transgelin-2 complex. Furthermore, molecular dynamics simulations indicated that residues Val^34^, Gly^35^, Ala^74^, and Gln^81^ may be key sites for activating transgelin-2.

Known for its amphiphilicity and low toxicity, glycyrrhizin can self-assemble into nanoparticles under specific conditions to enhance drug solubility. Through self-assembly simulation experiments, we revealed the formation mechanism of glycyrrhizin nanoparticles, and found that hydrogen bonding and hydrophobic interactions were the main driving forces for the formation of the nanoparticles, with the hydrophobic domain of glycyrrhizin located inside and its hydrophilic domain exposed outside.

Glycyrrhizin exhibited synergistic anti-asthmatic effects with salbutamol, but the functional and mechanistic understanding were unclear. We simulated the clinical dosages of 8.31 mmol/L salbutamol and 24.93 mmol/L glycyrrhizin in an aqueous environment. The study showed that most of the salbutamol molecules were attached to the surface of glycyrrhizin nanoparticles via hydrogen bonding and hydrophobic interactions, forming a new NDDS. Salbutamol may use the exposed hydrophilic domain of glycyrrhizin on the surface of NDDS to target transgelin-2 and deliver most of the drugs to the lesion area, releasing the drugs to exert synergistic anti-asthmatic effects in a special microenvironment.

Besides transgelin-2, some targets (i.g., ESR1, EGFR, CASP3, and SRC) may be important for the anti-asthmatic effect of glycyrrhizin, and some signaling pathways (i.g., SRC/EGFR signaling pathway) are also promising research directions for improving asthma.

## Data Availability

The original contributions presented in the study are included in the article/Supplementary Material, further inquiries can be directed to the corresponding author.
