# Supplementary material for: Micro-simulation insights into the functional and mechanistic understanding of glycyrrhizin against asthma
Source: Front Pharmacol. 2023 Aug 28;14:1220368. doi: 10.3389/fphar.2023.1220368 (PMC10497961; doi:10.3389/fphar.2023.1220368)
Supplement: Supplementary file 1 [file Table1.DOCX]

Supplementary Material

Micro-simulation insights into the functional and mechanistic understanding of glycyrrhizin against asthma

Jian-hong Qi^1,†^, Dong-chuan Xu^3,†^, Xiao-long Wang^2,4,5,†^, Ding-yuan Cai^1^, Yi Wang^1^, Wei Zhou^1,*^

^1^Department of Pharmaceutics, China Pharmaceutical University, Nanjing 211198, China.

^2^Shandong University of Traditional Chinese Medicine, Jinan 250355, China.

^3^Shandong Academy of Traditional Chinese Medicine, Jinan 250014, China.

^4^Key Laboratory of Traditional Chinese Medicine Classical Theory, Ministry of Education, Shandong University of Traditional Chinese Medicine, Jinan 250355, China.

^5^Shandong Provincial Key Laboratory of Traditional Chinese Medicine for Basic Research, Shandong University of Traditional Chinese Medicine, Jinan 250355, China.

**† These authors have contributed equally to this work.**

*** Correspondence:**

Corresponding authors:

Wei Zhou, professor

E-mail: zw_why@cpu.edu.cn

# Supplementary Figures


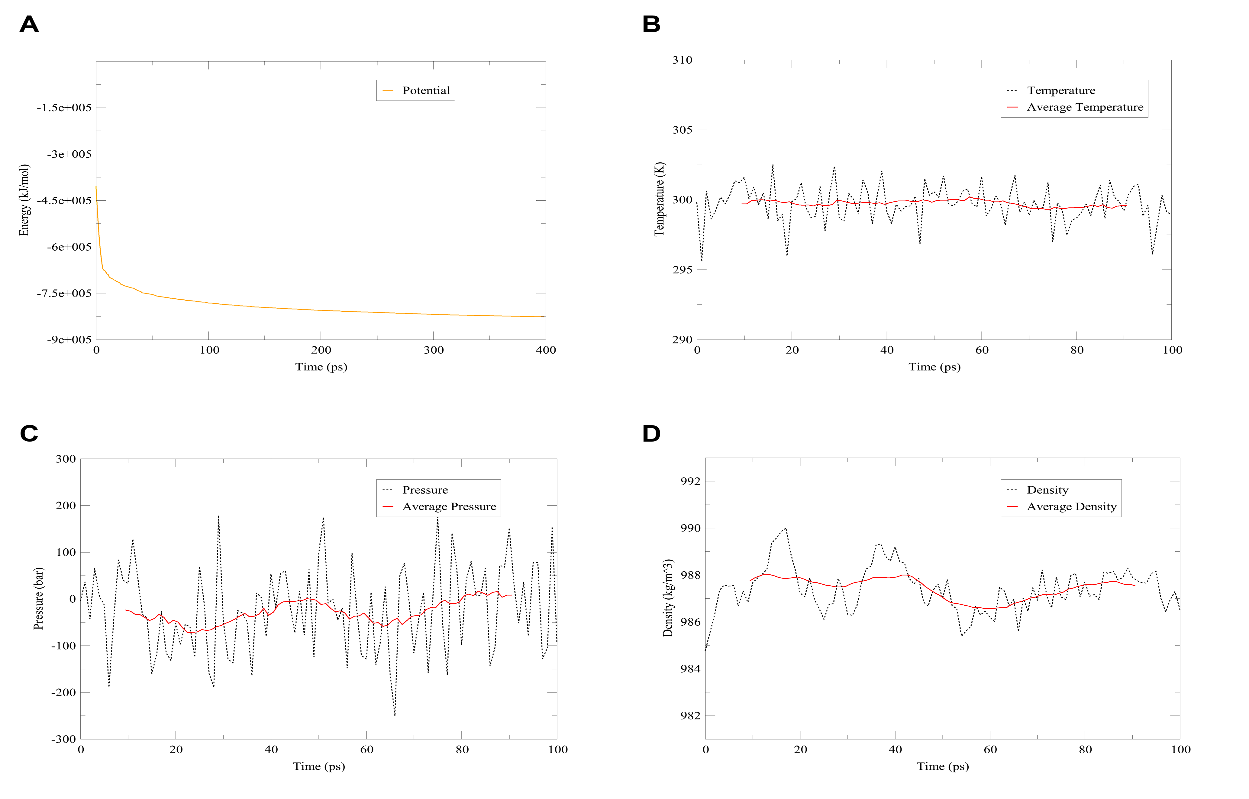


**Figure S1.** The pre-processing dynamics simulation of glycyrrhizin and transgelin-2

***Notes*: (A)** System energy minimization processing. **(B)** System temperature stabilization processing. **(C)** System pressure stabilization processing. **(D)** System density stabilization processing.


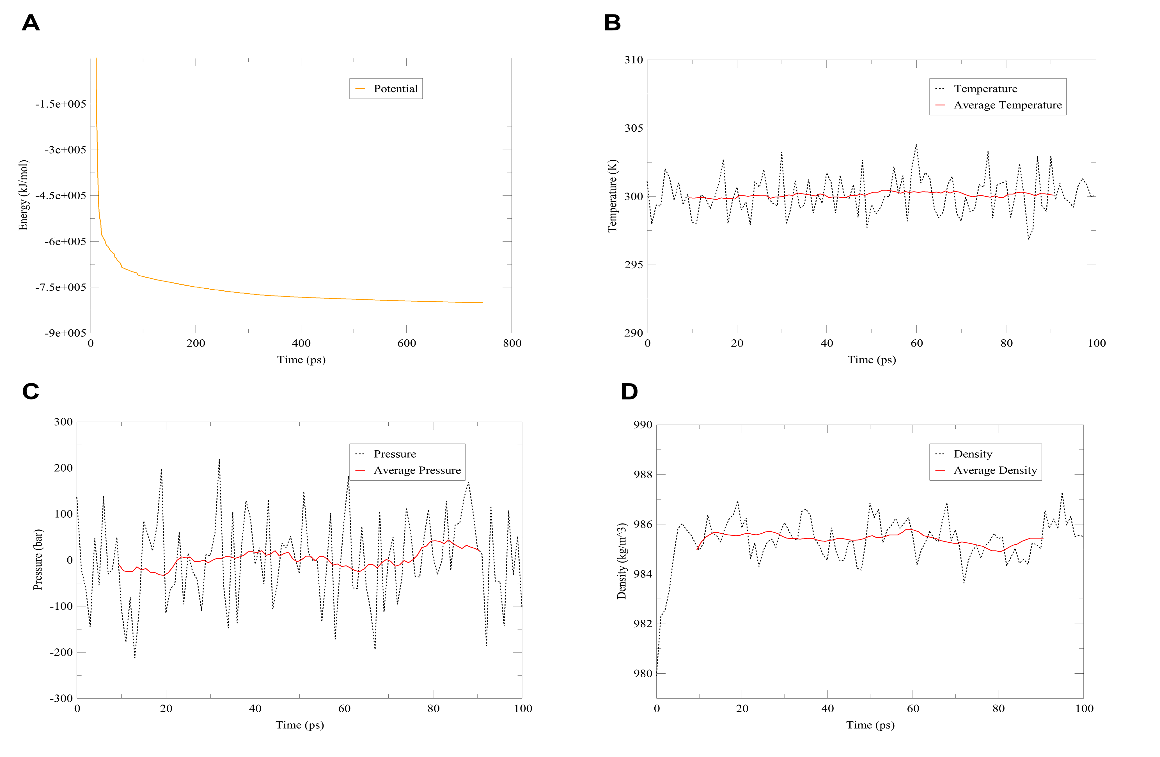


**Figure S2.** The pre-processing dynamics simulation of TSG12 and transgelin-2

***Notes*: (A)** System energy minimization processing. **(B)** System temperature stabilization processing. **(C)** System pressure stabilization processing. **(D)** System density stabilization processing.


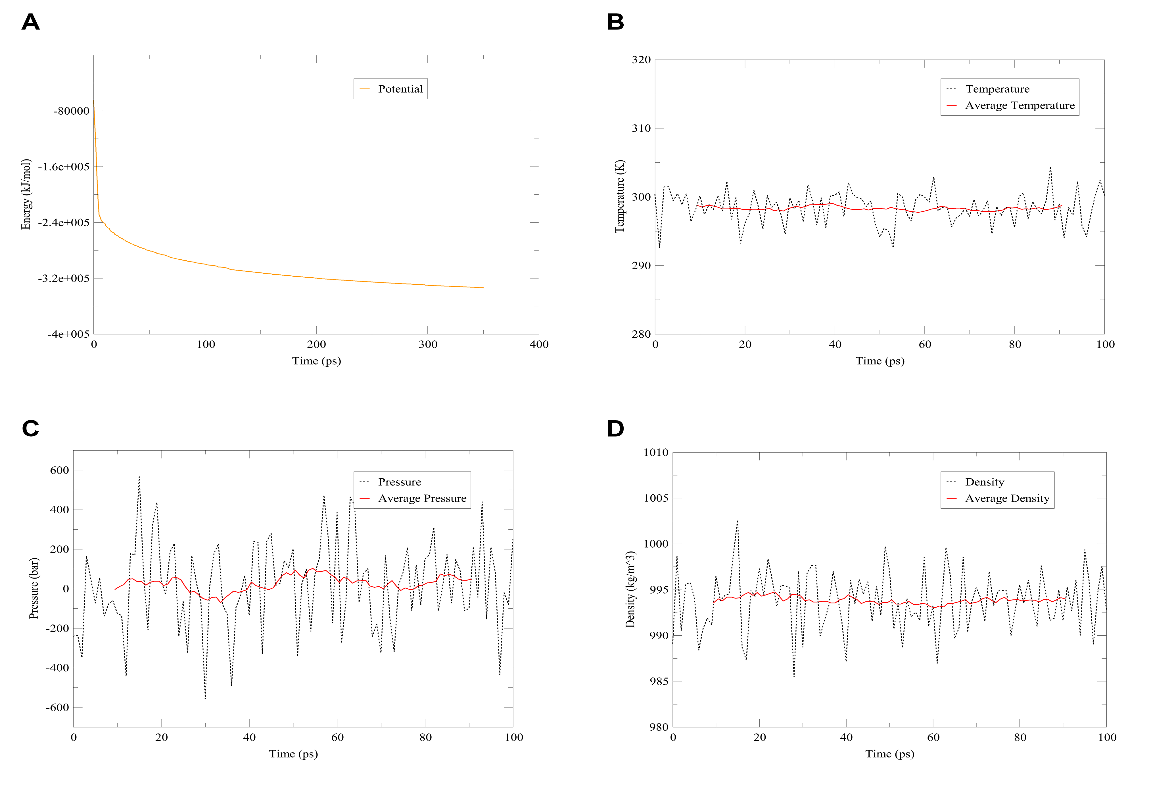


**Figure S3.** The pre-processing self-assembly simulation of glycyrrhizin

***Notes*: (A)** System energy minimization processing. **(B)** System temperature stabilization processing. **(C)** System pressure stabilization processing. **(D)** System density stabilization processing.


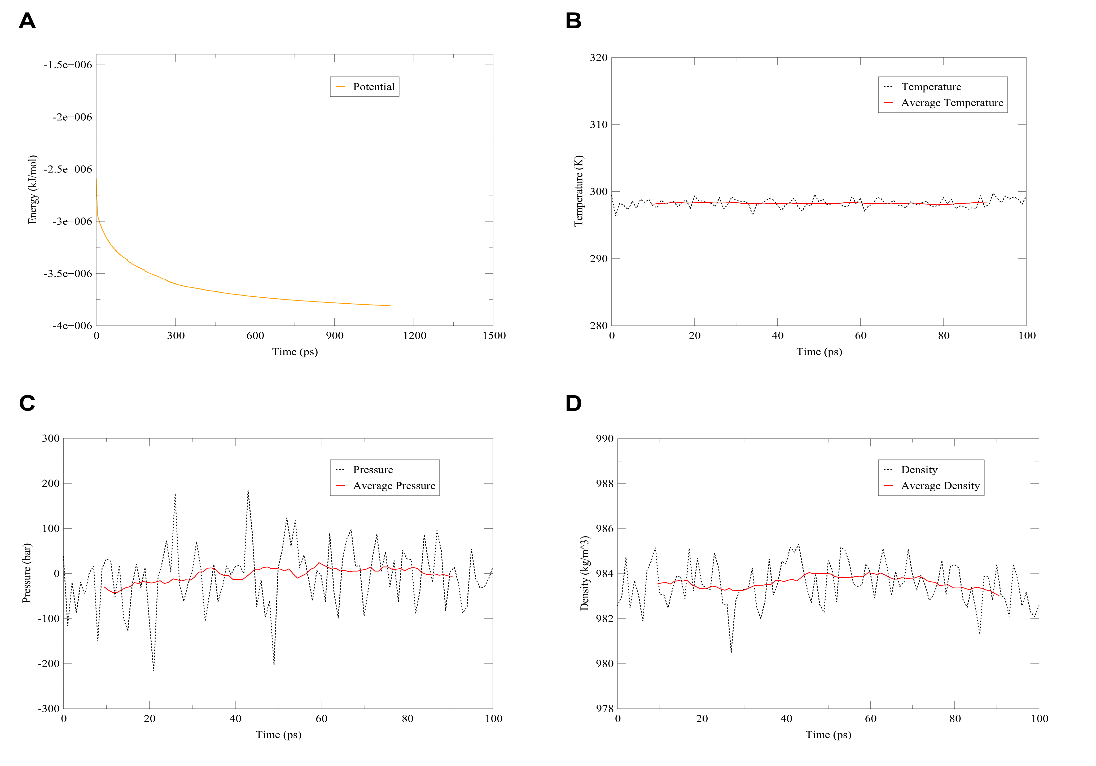


**Figure S4.** The pre-processing mechanistic simulation underlying the synergistic effects of glycyrrhizin and salbutamol against asthma

***Notes*: (A)** System energy minimization processing. **(B)** System temperature stabilization processing. **(C)** System pressure stabilization processing. **(D)** System density stabilization processing.

# Supplementary Tables

**Table S1.** Annotation information of GO.

| **Biological information** | **Enrichment** | ***p*-value** | **Count** | **Class** | **Log*P*** |
| --- | --- | --- | --- | --- | --- |
| regulation of kinase activity | 15.12055671 | 8.19E-13 | 7 | BP | -12.0868 |
| regulation of protein serine/threonine kinase activity | 23.14461066 | 9.36E-12 | 5 | BP | -11.029 |
| regulation of protein kinase activity | 14.96478175 | 7.66E-11 | 6 | BP | -10.116 |
| positive regulation of kinase activity | 18.50631824 | 8.36E-11 | 5 | BP | -10.0777 |
| regulation of defense response | 14.78872549 | 8.68E-11 | 6 | BP | -10.0614 |
| positive regulation of transferase activity | 15.60088944 | 4.38722E-10 | 5 | BP | -9.35781 |
| regulation of MAPK cascade | 13.17308532 | 2.23365E-09 | 5 | BP | -8.65098 |
| protein tyrosine kinase activity | 39.7483531 | 8.21507E-09 | 6 | MF | -8.08539 |
| serine-type peptidase activity | 28.56912879 | 5.89786E-08 | 6 | MF | -7.22931 |
| endopeptidase activity | 15.0222649 | 3.3156E-07 | 7 | MF | -6.47944 |
| membrane raft | 16.82598996 | 1.31687E-06 | 6 | CC | -5.88046 |
| membrane microdomain | 16.77453433 | 1.34046E-06 | 6 | CC | -5.87275 |
| peptidase activity | 10.64806131 | 3.2754E-06 | 7 | MF | -5.48474 |
| protein kinase activity | 9.708447305 | 3.02663E-05 | 6 | MF | -4.51904 |
| cell projection membrane | 13.28796688 | 3.41802E-05 | 5 | CC | -4.46623 |
| leading edge membrane | 20.66016093 | 4.10269E-05 | 4 | CC | -4.38693 |
| phosphotransferase activity, alcohol group as acceptor | 8.162608225 | 7.92138E-05 | 6 | MF | -4.1012 |
| caveola | 33.44678492 | 9.95514E-05 | 3 | CC | -4.00195 |
| kinase activity | 7.534715285 | 0.000122829 | 6 | MF | -3.9107 |
| lytic vacuole | 7.352912503 | 0.000140318 | 6 | CC | -3.85289 |
| lysosome | 7.352912503 | 0.000140318 | 6 | CC | -3.85289 |

**Table S2.** Annotation information of KEGG.

| **Pathway** | **Enrichment** | ***p*-value** | **Count** | **Class** | **Log*P*** |
| --- | --- | --- | --- | --- | --- |
| Adherens junction | 64.3811353 | 1.41028E-08 | 5 | KEGG | -7.850693415 |
| PI3K-Akt signaling pathway | 18.07764081 | 9.48555E-08 | 7 | KEGG | -7.022937677 |
| Rap1 signaling pathway | 26.12034632 | 1.00265E-07 | 6 | KEGG | -6.998849488 |
| Fluid shear stress and atherosclerosis | 32.8853281 | 4.13178E-07 | 5 | KEGG | -6.383862508 |
| MAPK signaling pathway | 18.65739023 | 7.22485E-07 | 6 | KEGG | -6.141170943 |
| Epithelial cell signaling in Helicobacter pylori infection | 52.24069264 | 1.03338E-06 | 4 | KEGG | -5.98574112 |
| Central carbon metabolism in cancer | 52.24069264 | 1.03338E-06 | 4 | KEGG | -5.98574112 |
| Lipid and atherosclerosis | 21.260747 | 3.53943E-06 | 5 | KEGG | -5.451067036 |
| Parathyroid hormone synthesis, secretion and action | 34.49857061 | 5.4459E-06 | 4 | KEGG | -5.263930665 |
| Ras signaling pathway | 19.45132173 | 5.45874E-06 | 5 | KEGG | -5.262907596 |
| EGFR tyrosine kinase inhibitor resistance | 34.716916 | 8.90925E-05 | 3 | KEGG | -4.050158741 |
| Coronavirus disease - COVID-19 | 15.76227795 | 0.000117031 | 4 | KEGG | -3.931699656 |
| Calcium signaling pathway | 15.23686869 | 0.000133322 | 4 | KEGG | -3.875098302 |
| Endocrine resistance | 27.98608534 | 0.000168948 | 3 | KEGG | -3.772246586 |
| HIF-1 signaling pathway | 25.1618015 | 0.000231293 | 3 | KEGG | -3.635837612 |
| Relaxin signaling pathway | 21.260747 | 0.000379375 | 3 | KEGG | -3.420931241 |
| Phospholipase D signaling pathway | 18.53132678 | 0.000566579 | 3 | KEGG | -3.246739589 |
| Oxytocin signaling pathway | 17.80932704 | 0.000635985 | 3 | KEGG | -3.196553097 |
| Focal adhesion | 13.64495703 | 0.001371844 | 3 | KEGG | -2.862695271 |
| Regulation of actin cytoskeleton | 12.58090075 | 0.002405177 | 3 | KEGG | -2.761885289 |

**Table S3.** The parameter information of ingredient-target-signaling pathway network.

| **Name** | **Degree** | **AverageShortest**  **PathLength** | **Closeness**  **Centrality** | **Betweenness**  **Centrality** | **Topological**  **Coefficient** | **Radiality** |
| --- | --- | --- | --- | --- | --- | --- |
| ASTHMA | 32 | 1.39622642 | 0.716216 | 0.504018 | 0.15625 | 0.900943 |
| Glycyrrhizin | 22 | 1.77358491 | 0.56383 | 0.182236 | 0.212121 | 0.806604 |
| EGFR | 20 | 1.66037736 | 0.602273 | 0.19091 | 0.172581 | 0.834906 |
| SRC | 13 | 1.9245283 | 0.519608 | 0.094994 | 0.208437 | 0.768868 |
| MET | 12 | 1.96226415 | 0.509615 | 0.059703 | 0.239247 | 0.759434 |
| FGFR1 | 11 | 2 | 0.5 | 0.053423 | 0.255132 | 0.75 |
| NOS3 | 9 | 2.0754717 | 0.481818 | 0.052189 | 0.268817 | 0.731132 |
| INSR | 9 | 2.0754717 | 0.481818 | 0.037056 | 0.286738 | 0.731132 |
| KIT | 8 | 2.11320755 | 0.473214 | 0.029146 | 0.310484 | 0.721698 |
| PI3K-Akt signaling pathway | 7 | 2.33962264 | 0.427419 | 0.013975 | 0.442177 | 0.665094 |
| Rap1 signaling pathway | 6 | 2.37735849 | 0.420635 | 0.006749 | 0.531746 | 0.65566 |
| MAPK signaling pathway | 6 | 2.41509434 | 0.414063 | 0.007182 | 0.491667 | 0.646226 |
| CASP3 | 5 | 2.22641509 | 0.449153 | 0.014821 | 0.412903 | 0.693396 |
| Adherens junction | 5 | 2.41509434 | 0.414063 | 0.004474 | 0.571429 | 0.646226 |
| Ras signaling pathway | 5 | 2.49056604 | 0.401515 | 0.00336 | 0.578947 | 0.627358 |
| Fluid shear stress and atherosclerosis | 5 | 2.64150943 | 0.378571 | 0.009615 | 0.32 | 0.589623 |
| Lipid and atherosclerosis | 5 | 2.56603774 | 0.389706 | 0.008353 | 0.329412 | 0.608491 |
| MMP3 | 4 | 2.26415094 | 0.441667 | 0.01128 | 0.475806 | 0.683962 |
| Epithelial cell signaling in Helicobacter pylori infection | 4 | 2.45283019 | 0.407692 | 0.003891 | 0.547619 | 0.636792 |
| Central carbon metabolism in cancer | 4 | 2.52830189 | 0.395522 | 0.002063 | 0.618421 | 0.617925 |
| Calcium signaling pathway | 4 | 2.45283019 | 0.407692 | 0.003146 | 0.571429 | 0.636792 |
| Coronavirus disease - COVID-19 | 4 | 2.49056604 | 0.401515 | 0.006477 | 0.325 | 0.627358 |
| Parathyroid hormone synthesis, secretion and action | 4 | 2.52830189 | 0.395522 | 0.008849 | 0.407895 | 0.617925 |
| IL2 | 3 | 2.30188679 | 0.434426 | 0.005835 | 0.430108 | 0.674528 |
| NQO1 | 3 | 2.30188679 | 0.434426 | 0.005458 | 0.602151 | 0.674528 |
| ACE | 3 | 2.30188679 | 0.434426 | 0.005594 | 0.591398 | 0.674528 |
| ESR1 | 3 | 2.30188679 | 0.434426 | 0.006454 | 0.580645 | 0.674528 |
| EGFR tyrosine kinase inhibitor resistance | 3 | 2.49056604 | 0.401515 | 0.001328 | 0.666667 | 0.627358 |
| Endocrine resistance | 3 | 2.49056604 | 0.401515 | 0.00318 | 0.52381 | 0.627358 |
| HIF-1 signaling pathway | 3 | 2.49056604 | 0.401515 | 0.001947 | 0.555556 | 0.627358 |
| Relaxin signaling pathway | 3 | 2.49056604 | 0.401515 | 0.001817 | 0.619048 | 0.627358 |
| Phospholipase D signaling pathway | 3 | 2.56603774 | 0.389706 | 0.001278 | 0.596491 | 0.608491 |
| Oxytocin signaling pathway | 3 | 2.49056604 | 0.401515 | 0.001817 | 0.619048 | 0.627358 |
| Focal adhesion | 3 | 2.49056604 | 0.401515 | 0.001328 | 0.666667 | 0.627358 |
| Regulation of actin cytoskeleton | 3 | 2.49056604 | 0.401515 | 0.001581 | 0.650794 | 0.627358 |
| PDE4B | 2 | 2.33962264 | 0.427419 | 0.00364 | 0.548387 | 0.665094 |
| GSTP1 | 2 | 2.33962264 | 0.427419 | 0.003056 | 0.564516 | 0.665094 |
| GSTM1 | 2 | 2.33962264 | 0.427419 | 0.003056 | 0.564516 | 0.665094 |
| PPARG | 2 | 2.33962264 | 0.427419 | 0.002756 | 0.564516 | 0.665094 |
| PDE4D | 2 | 2.33962264 | 0.427419 | 0.00364 | 0.548387 | 0.665094 |
| MIF | 2 | 2.33962264 | 0.427419 | 3.35E-04 | 0.83871 | 0.665094 |
| TGM3 | 2 | 2.33962264 | 0.427419 | 3.35E-04 | 0.83871 | 0.665094 |
| MMP8 | 2 | 2.33962264 | 0.427419 | 3.35E-04 | 0.83871 | 0.665094 |
| ANXA5 | 2 | 2.33962264 | 0.427419 | 3.35E-04 | 0.83871 | 0.665094 |
| RORA | 2 | 2.33962264 | 0.427419 | 3.35E-04 | 0.83871 | 0.665094 |
| STS | 2 | 2.33962264 | 0.427419 | 3.35E-04 | 0.83871 | 0.665094 |
| DPP4 | 2 | 2.33962264 | 0.427419 | 3.35E-04 | 0.83871 | 0.665094 |
| ALDH2 | 2 | 2.33962264 | 0.427419 | 3.35E-04 | 0.83871 | 0.665094 |
| ARSA | 2 | 2.33962264 | 0.427419 | 3.35E-04 | 0.83871 | 0.665094 |
| CBR1 | 2 | 2.33962264 | 0.427419 | 3.35E-04 | 0.83871 | 0.665094 |
| SHBG | 1 | 2.37735849 | 0.420635 | 0 | 0 | 0.65566 |
| PDE5A | 1 | 2.37735849 | 0.420635 | 0 | 0 | 0.65566 |
| CMA1 | 1 | 2.37735849 | 0.420635 | 0 | 0 | 0.65566 |
| MMP12 | 1 | 2.37735849 | 0.420635 | 0 | 0 | 0.65566 |
