# Supplementary figures and images for: Micro-simulation insights into the functional and mechanistic understanding of glycyrrhizin against asthma
Source: Front Pharmacol. 2023 Aug 28;14:1220368. doi: 10.3389/fphar.2023.1220368 (PMC10497961; doi:10.3389/fphar.2023.1220368)

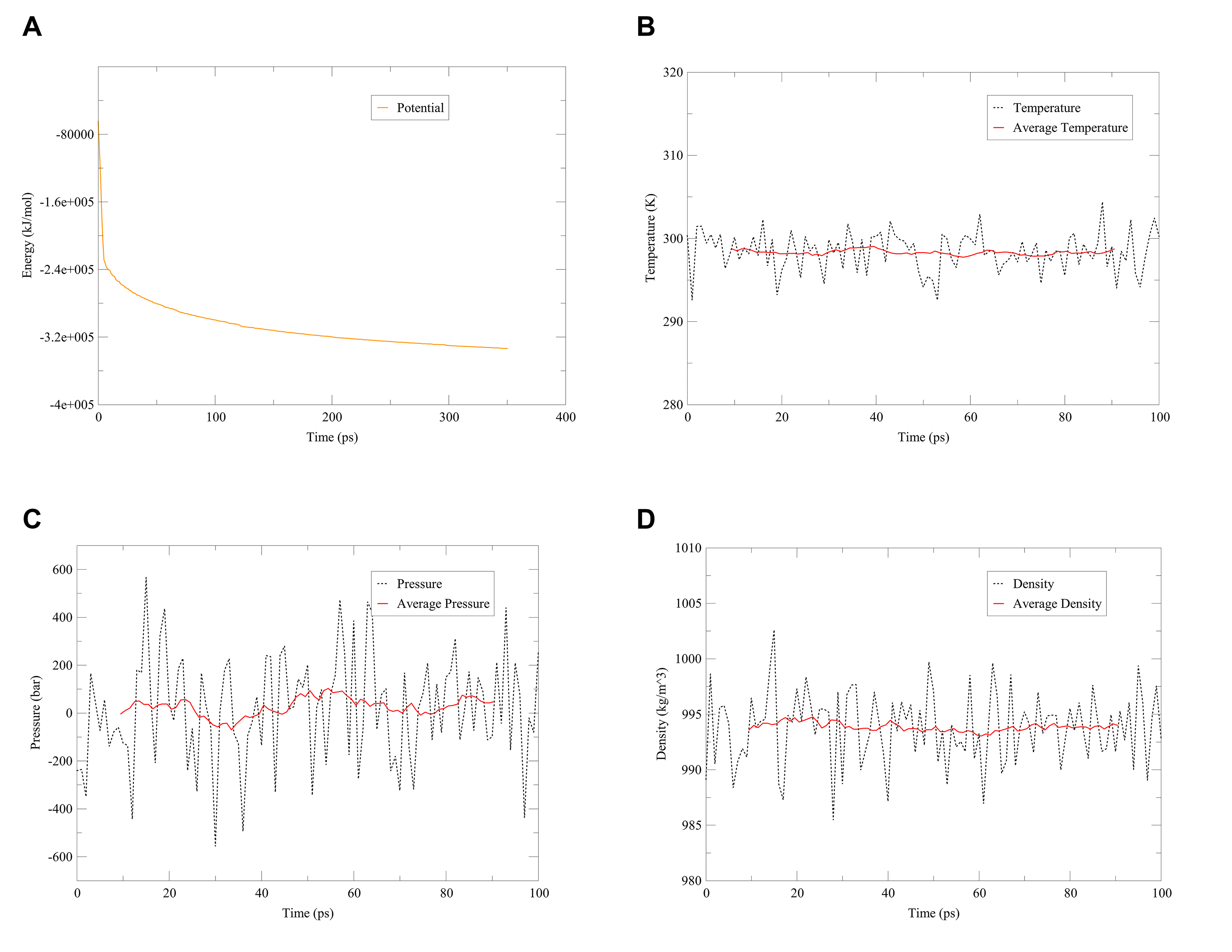

Supplement: Supplementary file 2 [file Image3.TIF]

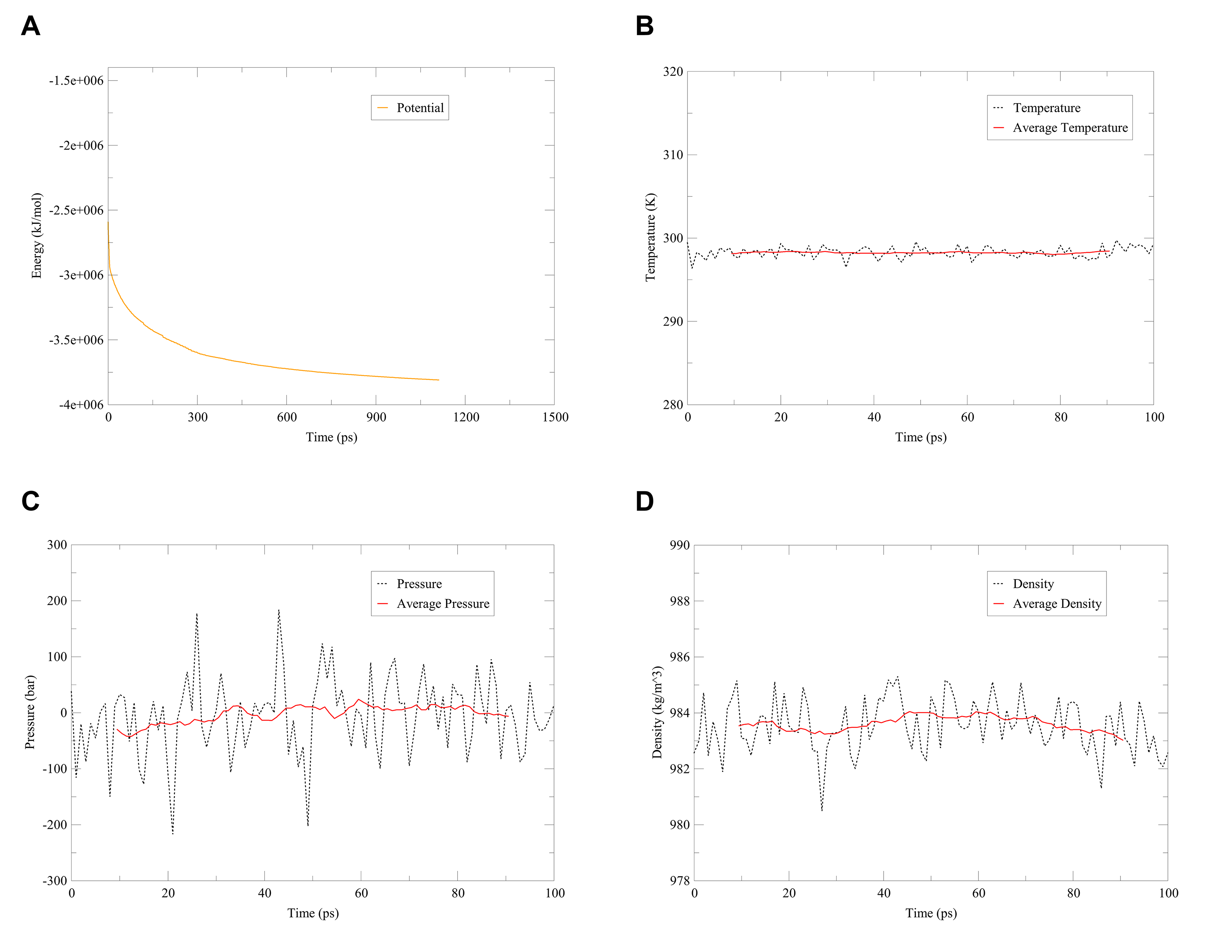

Supplement: Supplementary file 3 [file Image4.TIF]

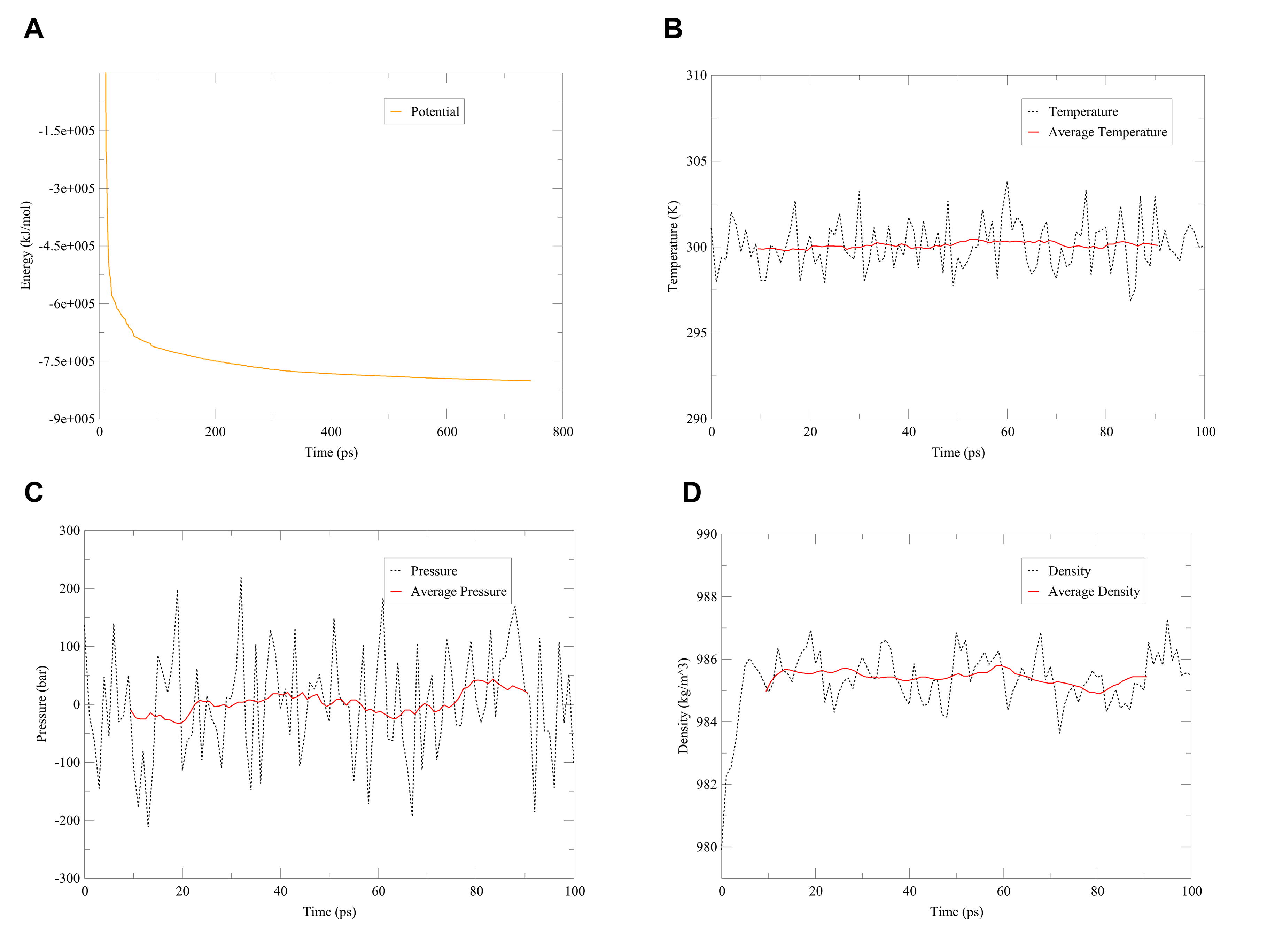

Supplement: Supplementary file 4 [file Image2.TIF]

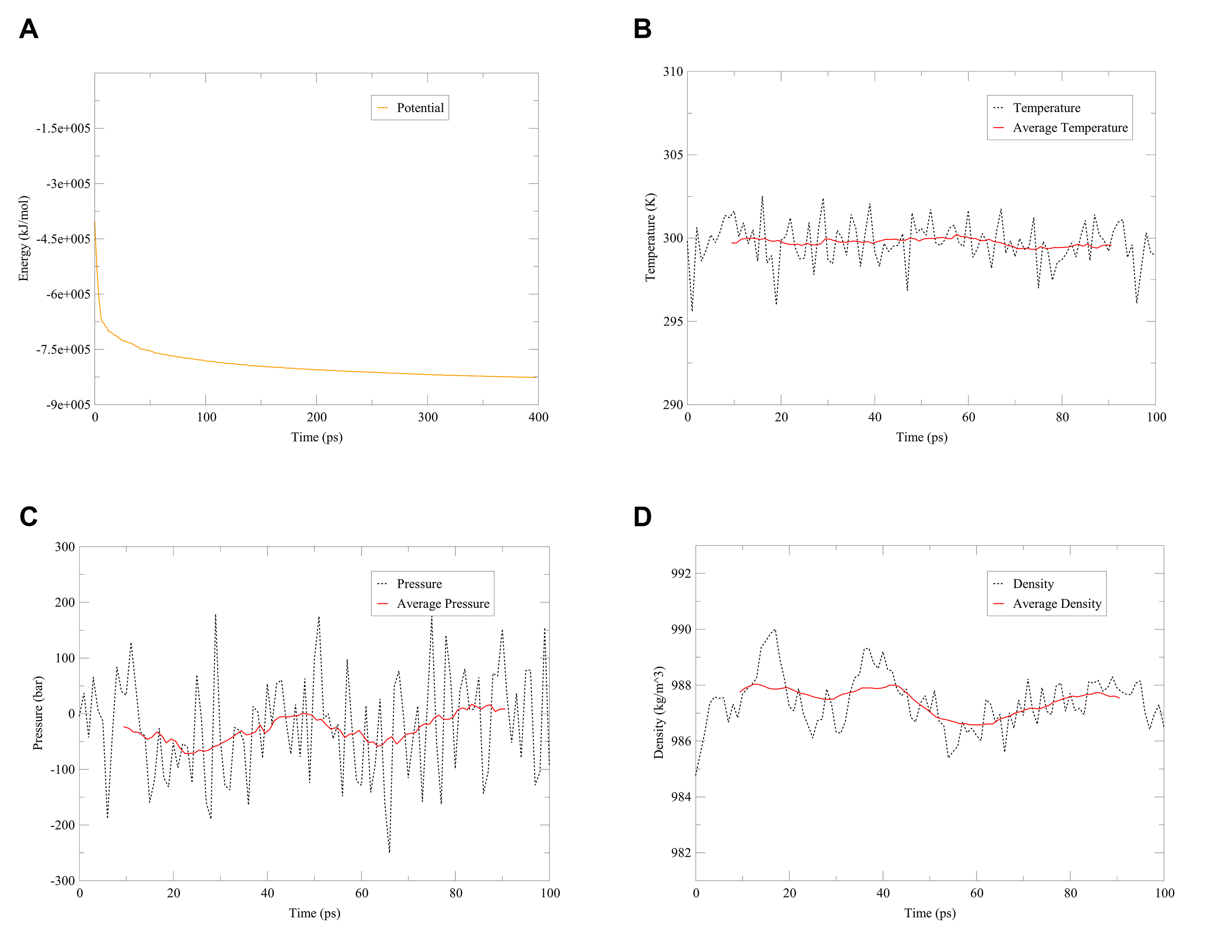

Supplement: Supplementary file 5 [file Image1.TIF]
